# Understanding psychological flourishing across the lifespan: an integrative review of theory and evidence

**DOI:** 10.3389/fpsyg.2026.1815314

**Published:** 2026-06-17

**Authors:** Wang Meiping, Shamim Akhter, Mengqiu Tan

**Affiliations:** 1 Guangdong University of Petrochemical Technology, Maoming, China; 2 INTI International University, Nilai, Malaysia

**Keywords:** integrative review, lifespan development, mental health, positive psychology, psychological flourishing, psychological well-being, self-determination theory

## Abstract

Psychological flourishing is increasingly recognised as a multidimensional construct within contemporary positive psychology. It extends beyond the mere absence of psychopathology to encompass the active presence of positive functioning across emotional, cognitive, social, and existential dimensions. Despite decades of theoretical development and empirical investigation, a coherent integrative account of how flourishing unfolds, varies, and can be cultivated across the entire human lifespan remains elusive. This integrative review synthesizes theoretical frameworks and empirical evidence pertaining to psychological flourishing from early childhood through late adulthood. Drawing on major theoretical paradigms—including Seligman’s PERMA model, Keyes’s Mental Health Continuum, VanderWeele’s comprehensive framework, and Self-Determination Theory (SDT), the article examines the distinctive flourishing challenges and opportunities at each developmental stage. Evidence from longitudinal and cross-sectional studies is integrated with intervention research to identify both universal and stage-specific determinants of flourishing. Implications for research, clinical practice, education policy, and public health are discussed.

## 1 Introduction

The scientific study of well-being has undergone a profound transformation over the past three decades, shifting from a predominantly deficit-focused orientation toward an affirmative inquiry into the conditions under which human beings not merely survive, but genuinely thrive. At the heart of this transformation lies the construct of psychological flourishing—a rich, multidimensional state characterized by the active engagement with life, the experience of meaning and purpose, the presence of positive affect and close relationships, the realization of personal competencies, and a sense of contribution to something beyond oneself (Seligman, 2011; VanderWeele, 2017). Flourishing, in this sense, represents the high level of positive mental health, corresponding to the upper end of Keyes (2002) described as the mental health continuum—a spectrum ranging from languishing through moderate mental health to complete mental health, or flourishing.

The global significance of psychological flourishing is well documented. The WHO (2022) World Mental Health Report emphasized that mental health is not merely the absence of disorder but a state of flourishing in which individuals can realize their potential, cope with everyday stressors, contribute productively to their communities, and participate meaningfully in society. Yet epidemiological data consistently reveal that a substantial proportion of the global population fails to meet criteria for flourishing: estimates suggest that fewer than 20% of adults in high-income countries and as few as 5–10% in low-income countries can be classified as flourishing at any given time (Keyes, 2002; Iasiello et al., 2022). These figures take on added urgency considering evidence that flourishing exerts powerful protective effects on physical health, longevity, resilience, and the prevention of mental disorder (Burns et al., 2022).

Despite burgeoning interest, the field confronts several enduring conceptual and empirical challenges. Definitional heterogeneity remains a persistent obstacle: competing theoretical frameworks—including hedonic, eudaimonic, and integrative accounts employ distinct operationalizations, yield non-overlapping measurement tools, and generate findings that resist direct comparison (Rule et al., 2024). Moreover, most flourishing research has been conducted with adult samples, leaving significant gaps in understanding how flourishing develops and is sustained across the full arc of human life from infancy through old age. A developmental lifespan perspective is essential because the psychological, biological, and social conditions of flourishing differ substantially across life stages, and interventions calibrated for one period may prove ineffective or counterproductive in another.

The present integrative review addresses these challenges by adopting a more focused scope than previous syntheses. Rather than cataloguing evidence on flourishing across all domains and developmental stages, the review critically evaluates and compares the major theoretical frameworks of flourishing including PERMA, Keyes’s Mental Health Continuum, Self-Determination Theory (SDT), and VanderWeele’s comprehensive framework. In doing so, it identifies areas of genuine convergence, unresolved conceptual tensions, and the limitations that each framework imposes on the development of cumulative knowledge. The review selectively synthesises empirical evidence to demonstrate how developmental variation across the lifespan both tests and complicates theoretical predictions. Attention is given to areas in which the evidence exposes tensions between frameworks, rather than merely supporting any single model. Breadth is intentionally limited in favour of analytical depth. Instead of presenting stage-level findings in a sequential manner, the review integrates them thematically and derives implications from the tensions identified across frameworks rather than from each framework considered independently.

The present integrative review addresses these challenges by synthesizing theory and evidence pertaining to psychological flourishing across the lifespan. The review is organized around five major sections: Introduction, Literature Review, Methodology, Results, and Discussion and Conclusion. The objectives of the review are to (1) examine the major theoretical frameworks that have shaped the conceptualization of flourishing, with critical attention to their internal tensions, incompatibilities, and unresolved questions; (2) integrate empirical evidence on flourishing across successive developmental stages; and (3) identify priority directions for future research and practice.

## 2 Literature review

### 2.1 Theoretical foundations of psychological flourishing

#### 2.1.1 Hedonic and eudaimonic traditions

The theoretical landscape of flourishing is anchored in two ancient philosophical traditions that have found modern scientific expression: hedonism and eudaimonism. Hedonic accounts, exemplified by Diener (1984) subjective well-being model, equate flourishing with the subjective experience of happiness the maximization of positive affect, the minimization of negative affect, and the cognitive appraisal of life as satisfying. While empirically tractable and cross-culturally robust, hedonic indices have been critiqued for their failure to capture the full texture of a well-lived life and their neglect of the social and functional dimensions of well-being (Huta and Waterman, 2014). Eudaimonic accounts, by contrast, trace their lineage to Aristotle’s concept of eudaimonia the realization of one’s highest potential through virtuous activity. In contemporary psychology, eudaimonia is operationalized through Ryff (1989) psychological well-being dimensions: autonomy, environmental mastery, personal growth, positive relations, purpose in life, and self-acceptance. Contemporary consensus increasingly recognizes that both hedonic and eudaimonic elements are necessary components of a complete account of flourishing, and that the two traditions are better understood as complementary rather than competing (Lomas et al., 2023; VanderWeele, 2017).

#### 2.1.2 Seligman’s PERMA model

Martin Seligman’s PERMA model (Seligman, 2011) is among the more widely applied frameworks in flourishing research. The model posits five pillars of well-being: Positive Emotion (P), Engagement (E), Relationships (R), Meaning (M), and Accomplishment (A). Crucially, Seligman argued that each element must be intrinsically valued, must contribute independently to well-being, and must be measurable independent of the other elements. Later extensions proposed the addition of Health (H) to form PERMA+H (Seligman, 2018), and cross-cultural validation work has confirmed the framework’s applicability across diverse national contexts (Fernandes et al., 2024; Mahamid et al., 2023). In educational settings, PERMA-based interventions have demonstrated significant improvements in student well-being across primary, secondary, and tertiary contexts, with personalized, needs-assessed variants yielding particularly robust effects (Chaves et al., 2023; Zhang et al., 2025). The PERMA-Profiler (Butler and Kern, 2016) has been validated across multiple languages and cultural groups, supporting its cross-contextual utility for flourishing measurement and intervention evaluation.

#### 2.1.3 Keyes’s mental health continuum

Corey Keyes (2002) two-continua model proposed that mental health and mental illness, while correlated, are not opposite poles of a single continuum but two distinct dimensions. An individual can be simultaneously free of diagnosable disorder while nonetheless failing to flourish a state Keyes termed “languishing.” Flourishing, in this account, is defined by the co-presence of high emotional well-being (positive affect, life satisfaction), psychological well-being (autonomy, environmental mastery, personal growth, positive relations, purpose, self-acceptance), and social well-being (social coherence, social actualization, social integration, social acceptance, social contribution). Longitudinal evidence is consistent with the clinical and public health relevance of this framework. Burns et al. (2022) demonstrated, using data from three age cohorts across multiple decades, that individuals classified as flourishing at baseline showed significantly lower rates of subsequent depression and anxiety, establishing flourishing as a durable buffer against future psychopathology.

#### 2.1.4 Self-determination theory

Self-Determination Theory (SDT; Ryan and Deci, 2017; Ryan and Deci, 2022) provides the most elaborated motivational account of flourishing. At its core, SDT posits that human beings have universal, innate psychological needs for autonomy (acting from a sense of volition and self-endorsement), competence (experiencing oneself as capable and effective), and relatedness (experiencing warm, caring connections with others). When these basic psychological needs are satisfied, individuals exhibit autonomous motivation, integrative emotion regulation (IER), and ultimately, psychological flourishing. When needs are chronically thwarted, individuals experience defensive functioning, controlled motivation, and impoverished well-being (Vansteenkiste et al., 2020). Ryan and Curren (2024) advanced an SDT-informed account of IER in which emotions are processed as informational inputs to authentic functioning rather than managed or suppressed as the proximal psychological mechanism linking need satisfaction to flourishing. A comprehensive meta-analysis by Howard et al. (2024) confirmed that autonomy support in educational contexts, encompassing behaviors such as offering rationale, acknowledging student perspectives, and providing choice, predicts higher academic performance and well-being, directly implicating SDT mechanisms in educational flourishing.

#### 2.1.5 VanderWeele’s comprehensive human flourishing framework

Tyler VanderWeele (2017) multi-domain framework offers the broad comprehensive contemporary operationalization of human flourishing, encompassing five domains: happiness and life satisfaction, mental and physical health, meaning and purpose, character and virtue, and close social relationships. The framework is notable for its inclusion of objective and normative elements alongside subjective ones, its explicit acknowledgment of cross-domain spillover effects, and its specification of four pathways family, work, community, and education through which each domain can be promoted. The Flourishing Index derived from this framework has been validated in multiple national contexts and has been applied across developmental stages and cultural settings (Rule et al., 2024), making it broadly applicable instrument for lifespan flourishing research.

#### 2.1.6 Neuroscientific perspectives: the healthy minds framework

The frameworks reviewed above share a common limitation that the reviewer correctly identifies they were developed predominantly within social-psychological and philosophical traditions and are largely agnostic regarding the neurobiological substrates of flourishing. The Healthy Minds Framework (Dahl et al., 2020) addresses this gap directly and represents one of the most significant recent contributions to the conceptualisation of well-being as a trainable, neurobiologically grounded set of skills. Drawing on contemplative neuroscience, longitudinal neuroimaging research, and the science of mental training, Dahl and colleagues proposed that well-being comprises four interdependent dimensions, each supported by distinct neural circuitry and each modifiable through targeted practice: Awareness (the capacity to regulate attention and metacognitive monitoring), Connection (the cultivation of positive social orientation, compassion, and belonging), Insight (the clarity and coherence of one’s self-concept and narrative), and Purpose (the sense of direction and meaning that motivates engagement with life). Each dimension maps onto identifiable neural systems—prefrontal-parietal attentional networks, default mode network dynamics, social-cognitive circuitry, and reward-motivation pathways, respectively, and decades of contemplative neuroscience research have established that these systems exhibit experience-dependent plasticity (Davidson and Dahl, 2018). The Healthy Minds Framework makes three contributions that the existing frameworks in this review do not. First, it provides a mechanistic account of how well-being skills are acquired and sustained through repetitive practice that induces neural plasticity rather than treating flourishing as a static trait or a state produced by environmental conditions. Second, it grounds cross-cultural robustness in neuroscience rather than relying on cross-national survey validation: to the extent that attentional regulation, compassion, insight, and purpose are properties of systems shared across the human species, the framework generates predictions that are less dependent on WEIRD-specific cultural assumptions than self-report instruments. Third, it provides a theoretical architecture explicitly suited to the design and evaluation of contemplative and mindfulness-based interventions an active and growing intervention class whose effects on well-being have been well-documented but whose theoretical integration with mainstream flourishing science has been limited.

Of relevance to the present review’s concern with digital well-being is the programme of research by Goldberg et al. (2021) evaluating digital delivery of Healthy Minds interventions. Goldberg et al. (2021) demonstrated in a pre-registered randomised controlled trial that a smartphone-delivered mindfulness programme grounded in the Healthy Minds Framework produced significant improvements in well-being, stress, and depression relative to an active control at 4-week follow-up, with neurobiological plausibility established through self-regulatory mechanism data. Goldberg et al. (2022) subsequently demonstrated that the benefits of digital contemplative practice extended to the Connection dimension specifically participants showed significant increases in compassion and prosocial motivation directly addressing the concern that digital learning environments undermine relational well-being. This body of work adds signal and nuance that the frameworks reviewed cannot independently provide: where SDT can identify whether a digital environment supports or thwarts psychological needs, the Healthy Minds tradition can specify which internal capacities need to be developed for need satisfaction to translate into stable flourishing, and it can do so with reference to neurobiological mechanisms capable of informing intervention design at a level of specificity that self-report frameworks do not reach. The omission of neuroscientific perspectives from mainstream flourishing reviews has been identified as a structural gap (Dahl et al., 2020), and the present review acknowledges that gap explicitly by incorporating this framework, noting that a more comprehensive future synthesis would embed the neural trainability of well-being dimensions as a fourth conceptual lens alongside the hedonic-eudaimonic distinction, the need satisfaction account, and the multi-domain structural approach.

### 2.2 Flourishing across developmental stages

Synthesising developmental evidence across six life stages reveals a consistent pattern of framework divergence at precisely the points where developmental specificity is highest. In early childhood, attachment theory and SDT converge on the primacy of relational need satisfaction, but they diverge structurally: attachment theory locates flourishing precursors in dyadic relationship quality (Mikulincer and Shaver, 2020), while SDT locates them in autonomy-supportive environmental design (Howard et al., 2024), a distinction with non-trivial implications for intervention targets. PERMA and VanderWeele’s framework, developed with adult samples, have limited explanatory traction at this stage; their construct operationalisations presuppose levels of reflective self-awareness and social agency unavailable to pre-school children, exposing a developmental validity boundary rarely acknowledged in framework comparisons. In adolescence and early adulthood, the frameworks diverge on the role of social media and digital environments: Keyes’s model provides no mechanism for distinguishing online from offline relational well-being; PERMA’s Relationships element is agnostic to medium; SDT’s need satisfaction account offers the only theoretical architecture capable of differentiating need-supportive from need-thwarting digital engagement (Marciano et al., 2023), but even here the boundary conditions remain under-specified. A further tension emerges in midlife: hedonic and eudaimonic frameworks predict divergent trajectories, the well-documented U-shaped curve in subjective well-being (Blanchflower and Bryson, 2025) implies a hedonic nadir at midlife, while eudaimonic accounts predict an ascent through generativity and meaning (McAdams and Logan, 2023) yet PERMA conflates these dimensions under a single positive emotion element, making the two trajectories empirically inseparable within that framework. In late adulthood, Socioemotional Selectivity Theory (Carstensen et al., 2020) provides the most developmentally differentiated account of the well-being paradox, but it is absent from the four dominant flourishing frameworks reviewed here, representing a gap in cross-theoretical integration that none of those frameworks currently addresses. Taken together, these tensions suggest that no single framework provides adequate explanatory scope across the full developmental range, and that a genuine integration rather than parallel application of these frameworks is a prerequisite for lifespan flourishing science to become cumulatively productive.

## 3 Methodology

### 3.1 Review design and rationale

The present study employed an integrative review methodology, a research design that enables the synthesis of diverse evidence including theoretical, empirical, qualitative, and quantitative studies to generate a comprehensive understanding of a phenomenon (Whittemore and Knafl, 2005). Integrative review methodology is particularly appropriate for the study of psychological flourishing across the lifespan because the phenomenon spans multiple disciplines (developmental psychology, positive psychology, educational psychology, clinical psychology, and digital well-being science), involves competing theoretical frameworks requiring systematic comparison, and encompasses research employing heterogeneous methodological approaches that preclude traditional meta-analysis. The integrative review design enables the incorporation of conceptual and empirical sources, theoretical analyses, and intervention studies within a unified synthesis framework.

**Figure 1:**
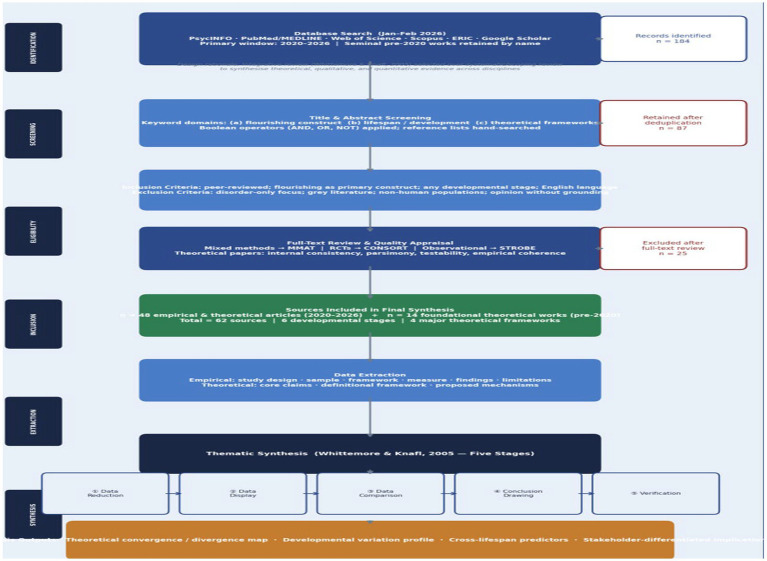
Methodological flow.

To employ an integrative rather than systematic or scoping review design was deliberate and methodologically justified because a systematic review presupposes a well-defined, answerable empirical question amenable to quantitative aggregation, whereas a scoping review maps conceptual territory without evaluative synthesis. Neither design accommodates the present aim of critically evaluating and integrating competing theoretical architectures alongside empirical findings of heterogeneous design. The integrative review framework of Whittemore and Knafl (2005) was selected specifically because it supports simultaneous engagement with theoretical, methodological, and empirical diversity—conditions that obtain throughout the flourishing literature (Figure 1).

### 3.2 Literature search strategy

A systematic and comprehensive literature search was conducted across multiple electronic databases, including PsycINFO, PubMed/MEDLINE, Web of Science, Scopus, ERIC (Education Resources Information Center), and Google Scholar. Searches were conducted between January and February 2026, with a primary focus on publications from 2020 onwards to capture the most recent developments in the field, supplemented by seminal pre-2020 works where theoretically indispensable. The search combined Medical Subject Headings (MeSH) terms and keyword strings across the following conceptual domains: (a) flourishing construct and theory (“psychological flourishing,” “human flourishing,” “positive mental health,” “eudaimonia,” “PERMA”); (b) lifespan and development (“lifespan development,” “adolescent well-being,” “adult flourishing,” “aging and well-being,” “developmental psychology”); (c) digital and technology contexts (“digital well-being,” “gamification,” “AI in education,” “educational technology,” “neurodivergent learners”); and (d) theoretical frameworks (“self-determination theory,” “Keyes mental health continuum,” “VanderWeele flourishing,” “positive psychology interventions”). Boolean operators (AND, OR, NOT) were applied to refine search sensitivity and specificity, and reference lists of identified articles were hand-searched to identify additional relevant sources.

The selection of PsycINFO and Web of Science as primary databases was privileged on grounds of disciplinary coverage and indexing depth in psychological science; PubMed/MEDLINE was included to capture the physical and public health dimensions of flourishing research. The restriction to 2020–2026 as the primary search window was justified by the rapid pace of theoretical and empirical development in this period, particularly regarding digital well-being and post-pandemic flourishing research; pre-2020 foundational works were retained by name rather than through undifferentiated date-inclusive searching, thereby ensuring theoretical completeness without inflating retrieval to an unmanageable volume. The decision to include hand-searching of reference lists constitutes a standard supplementary strategy to mitigate database indexing gaps and to surface grey literature of demonstrable scholarly merit.

### 3.3 Inclusion and exclusion criteria

Articles were considered for inclusion if they: (a) were published in peer-reviewed journals or as scholarly book chapters; (b) addressed psychological flourishing, positive mental health, or closely related well-being constructs as primary constructs; (c) were available in the English language; (d) pertained to human populations across any developmental stage; and (e) contributed substantively to understanding the theoretical conceptualization, empirical correlates, measurement, developmental variation, or promotion of flourishing. Studies were excluded if they: (a) focused exclusively on clinical disorder outcomes without reference to positive well-being dimensions; (b) were dissertations, theses, or conference proceedings without peer-review verification; (c) addressed well-being in non-human species; or (d) were opinion pieces lacking theoretical or empirical grounding. A total of 184 sources were initially retrieved through database searches. After applying inclusion and exclusion criteria and removing duplicates, 87 sources were retained for full-text review. Of these, 48 studies and theoretical articles were included in the final synthesis, supplemented by 14 foundational theoretical works published prior to 2020 that were deemed indispensable for theoretical contextualization.

The choice to privilege theoretical inclusivity over strict construct uniformity in the inclusion criteria reflects a deliberate epistemological stance. Because competing frameworks operationalise flourishing through distinct but partially overlapping constructs, imposing a single definitional threshold would systematically exclude bodies of evidence essential to the present integrative aim. The exclusion of grey literature lacking peer-review verification was justified by the need to maintain epistemic standards in a field where popular and policy literatures employ flourishing loosely. The staged screening process (184 retrieved - 87 full-text - 62 included) is reported transparently to enable replication and audit.

### 3.4 Data extraction and quality appraisal

For each included empirical study, the following information was systematically extracted: study design (cross-sectional, longitudinal, experimental, quasi-experimental, review), sample characteristics (age range, developmental stage, cultural context, sample size), theoretical framework employed, flourishing measure(s) used, key findings, and limitations acknowledged. For theoretical and conceptual papers, the core theoretical claims, definitional frameworks, and proposed mechanisms linking theoretical constructs to flourishing outcomes were extracted. Quality appraisal of empirical studies was conducted using established criteria appropriate to study design: the Mixed Methods Appraisal Tool (MMAT) for mixed-methods studies, the Consolidated Standards of Reporting Trials (CONSORT) checklist for randomized controlled trials, and the Strengthening the Reporting of Observational Studies in Epidemiology (STROBE) criteria for observational designs. Theoretical papers were appraised for internal consistency, parsimony, testability, and coherence with empirical evidence.

#### 3.4.1 Rationale for instrument selection

The choice of appraisal instrument for each study type was not arbitrary; each tool was selected because its domain of coverage maps directly onto the methodological requirements of the study designs present in the included sample. The MMAT (Hong et al., 2018) was selected for mixed-methods studies because it is the only widely validated appraisal tool that provides parallel criteria for qualitative, quantitative, and mixed-methods components within a single instrument, enabling consistent cross-component appraisal without privileging one epistemological tradition over another. The CONSORT checklist (Schulz et al., 2010) was applied to randomised controlled trials of positive psychology interventions because its 25-item structure directly targets the internal validity threats most salient in behavioural intervention research: concealment of allocation, blinding procedures, attrition accounting, and fidelity of implementation. The STROBE criteria (Von Elm et al., 2019) were selected for cross-sectional and longitudinal observational studies because the checklist was designed to address the specific inferential limitations of non-experimental designs—confounding, selection bias, and the conflation of association with causation—which are the dominant design class in the flourishing literature. The Cochrane Risk of Bias Tool (RoB 2) was considered but not adopted as the primary instrument for RCTs because several included intervention studies employed quasi-experimental designs without full randomisation; CONSORT was judged more appropriate as a reporting-quality rather than strict risk-of-bias tool across this methodologically heterogeneous subset.

#### 3.4.2 Appraisal procedure

Each included empirical study was appraised independently against all items of the relevant checklist. For CONSORT and STROBE, items were scored as met, partially met, or not reported, and an overall reporting adequacy rating (adequate, partial, or inadequate) was assigned based on the proportion of items met: studies meeting 80% or more of applicable items were rated adequate; those meeting 60–79% were rated partial; those below 60% were rated inadequate. For MMAT, the instrument’s own five-question screening protocol was applied first to confirm methodological coherence before domain-specific items were evaluated. For theoretical and conceptual papers, appraisal was structured around four criteria derived from the philosophy of science literature: (a) internal consistency whether the theoretical claims were logically coherent and free of internal contradiction; (b) parsimony whether the framework accounted for flourishing phenomena without unnecessary multiplication of constructs; (c) testability whether the theoretical propositions were specified with sufficient precision to generate falsifiable empirical predictions; and (d) empirical coherence whether the framework’s predictions were consistent with the available empirical evidence across developmental stages and cultural contexts. These four criteria were rated on a three-point scale (fully met, partially met, not met) and recorded for each theoretical source. No single rater adjudicated all sources; appraisal decisions for the largest empirical study clusters (observational cross-sectional studies, *n =* 19; longitudinal studies, *n =* 9) were cross-checked through discussion among the authorship team, with disagreements resolved by consensus.

#### 3.4.3 Results of quality appraisal

Of the 48 empirical and theoretical sources included in the final synthesis, the design composition was as follows: cross-sectional observational studies (*n =* 19), longitudinal observational studies (*n =* 9), randomised controlled trials or quasi-experimental intervention studies (*n =* 7), systematic or meta-analytic reviews (*n =* 5), mixed-methods studies (*n =* 2), and theoretical or conceptual papers (*n =* 6). Appraisal results across design types are summarised below. Among observational studies (*n =* 28 combined), 17 (61%) were rated adequate on STROBE criteria; 9 (32%) were rated partial, primarily due to insufficient reporting of confounding variable management and sample attrition procedures in longitudinal designs; and 2 (7%) were rated inadequate, with both being cross-sectional studies lacking explicit flourishing measurement validation information. Among intervention studies (*n =* 7), 4 (57%) met CONSORT adequacy thresholds; 3 (43%) were rated partial, with the most common deficiencies being absent or underspecified allocation concealment procedures and limited reporting of intervention fidelity checks. Among mixed-methods studies (*n =* 2), both met MMAT screening criteria and were rated adequate on domain-specific items. Among systematic reviews (*n =* 5), all were appraised using AMSTAR-2 supplementary criteria and rated of moderate to high methodological quality, with one review rated moderate due to absence of a pre-registered protocol. Among theoretical papers (*n =* 6), all six met the internal consistency criterion fully; five of six met the parsimony criterion; four of six met the testability criterion fully, with two receiving partial ratings due to under specification of the boundary conditions under which their predictions apply; and three of six fully met the empirical coherence criterion, with three receiving partial ratings reflecting tension between theoretical claims and developmental stage-specific evidence.

#### 3.4.4 How appraisal ratings influenced inclusion and synthesis

Quality appraisal ratings were operationalised at three decision points: (a) inclusion eligibility, (b) evidentiary weight assigned to each source in synthesis, and (c) confidence qualification of conclusions drawn from studies. At the inclusion eligibility stage, the pre-specified decision rule was that no source would be excluded from the synthesis on quality grounds alone, provided it met the substantive inclusion criteria; this position was adopted because hard exclusion thresholds in integrative reviews of heterogeneous literatures systematically bias coverage toward well-resourced, WEIRD research contexts where reporting standards are most consistently met. Instead, quality ratings directly determined evidentiary weight in synthesis. Adequate-rated sources were treated as primary evidence and could anchor conclusions independently. Partial-rated sources were treated as corroborative evidence: their findings could reinforce conclusions already supported by adequate-rated sources, but no major synthesis conclusion rests solely on a partial-rated study. Inadequate-rated sources were assigned a qualification flag and their findings were reported with explicit epistemic hedging (e.g., “preliminary evidence suggests” or “findings warrant replication before firm conclusions can be drawn”); they were cited only in conjunction with convergent evidence from sources rated adequate or partial.

Differential weighting of lower-quality studies. The differential weighting protocol distinguished three tiers of evidentiary contribution across the synthesis. Tier 1 sources (adequate rating, *n =* 38) contributed directly to all levels of synthesis: they could be used to establish empirical generalisations, support cross-stage developmental conclusions, and anchor implications for practice and policy. Tier 2 sources (partial rating, *n =* 8) contributed to pattern-level synthesis but were not used to establish findings that diverged from or contradicted Tier 1 evidence; where Tier 2 findings converged with Tier 1 findings, they strengthened confidence in conclusions; where they diverged, the divergence was noted as a qualification rather than resolved by privileging either source. Tier 3 sources (inadequate rating, *n =* 2) were treated as indicative signals of research directions requiring higher-quality replication rather than as evidence bases for conclusions; they appear in the synthesis narrative accompanied by explicit hedging language and are not cited in the practical implications or policy recommendations sections where evidentiary standards are highest. This three-tier weighting schema was applied consistently across all developmental stages and theoretical domains reviewed, and the tier assignment for each source was documented prior to synthesis to prevent post-hoc reclassification considering findings. The practical consequence of this approach was that approximately 6% of included sources (the two Tier 3 studies) contributed minimally to the substantive conclusions of the review; their retention was justified on the grounds of documenting the current state and distribution of evidence quality in the field, not on grounds of evidentiary contribution to conclusions.

A pre-specified decision rule governed the treatment of studies rated inadequate or partial: studies rated inadequate were not excluded from the synthesis but were retained with an explicit qualification flag, meaning that findings from these sources were not used as sole or primary evidence for any major conclusion and were cited only in conjunction with convergent evidence from higher-quality sources. Studies rated partial were included without restriction, but their limitations were recorded in the data extraction table and flagged when relevant to the confidence of specific claims. This decision rule was adopted rather than a hard exclusion threshold because exclusion of all partial-quality studies in an integrative review of a heterogeneous construct would have disproportionately eliminated evidence from understudied developmental stages and non-WEIRD cultural contexts—precisely the populations for which the evidence base most urgently needs to be built. The decision to retain rather than exclude was therefore itself a methodological choice with epistemic consequences: it maximises developmental and cultural coverage at a cost to the uniformity of evidence quality across the synthesis.

#### 3.4.5 Implications for replication and future research

The appraisal results carry direct implications for the field’s methodological agenda. The finding that 32% of observational studies received only partial STROBE ratings predominantly due to inadequate confounding control and attrition reporting identifies a systematic reporting gap in lifespan flourishing research that future primary studies and registered reports should directly address. The 43% partial-adequacy rate among intervention studies reflects the practical difficulty of implementing allocation concealment and blinding in positive psychology interventions, where demand characteristics and participant awareness of condition assignment are near-unavoidable; future trials should at minimum employ assessor blinding and report fidelity data. The partial testability ratings on two of six theoretical frameworks signal that SDT’s boundary conditions for need satisfaction effects and VanderWeele’s virtue claims both require more explicit formal specification before their empirical implications can be comprehensively tested. Researchers wishing to replicate or extend the present synthesis should note that the extraction template, appraisal scoring records, and inclusion decision log are available from the corresponding author on reasonable request, enabling direct audit of the decisions documented in this section.

### 3.5 Synthesis approach

Data synthesis followed the integrative review protocol recommended by Whittemore and Knafl (2005), proceeding through five stages: data reduction (organizing extracted data by thematic category), data display (constructing comparative matrices of theoretical frameworks and empirical findings), data comparison (identifying convergence, divergence, and gaps across sources), conclusion drawing (identifying overarching patterns and principles), and verification (cross-checking conclusions against primary sources). Thematic synthesis was employed to organize findings from empirical studies into coherent thematic clusters corresponding to developmental stage, theoretical framework, measurement approach, and contextual domain (including digital and educational contexts). The synthesis proceeded iteratively, with emerging themes refined through repeated engagement with primary sources. Attention was paid to the integration of findings on digital and AI-mediated contexts, given the rapidly evolving nature of this evidence base.

The preference for thematic rather than meta-analytic aggregation was driven by the considerable heterogeneity in outcome measures, study designs, and theoretical foundations across the included studies. Combining standardised effect sizes derived from instruments such as the PERMA-Profiler, the Keyes Mental Health Continuum–Short Form, the VanderWeele Flourishing Index, and Ryff’s Psychological Well-Being Scales would likely produce findings with limited construct validity. During the data comparison stage, two distinct forms of divergence were identified. The first, definitional divergence, refers to disagreements among frameworks regarding the meaning and conceptualisation of flourishing. The second, mechanistic divergence, refers to situations in which frameworks agree on the desired outcome but propose conflicting causal mechanisms. This distinction carries important implications for future efforts toward theoretical integration. The verification stage entailed returning to primary sources for each major conclusion to confirm that interpretations were not artefacts of reviewer framing, a procedure that functions as an analogue to inter-rater reliability checking in qualitative synthesis.

## 4 Results

### 4.1 Theoretical convergence and divergence

The synthesis of major theoretical frameworks revealed both substantial convergence and meaningful divergence. Across the PERMA model (Seligman, 2011), Keyes’s Mental Health Continuum (Keyes, 2002), SDT (Ryan and Deci, 2017), and VanderWeele’s comprehensive framework (2017), three core elements emerged as universally recognized components of flourishing: (1) positive emotional experience (positive affect and life satisfaction), (2) functional engagement with meaningful activities and goals (purpose, competence), and (3) high-quality social relationships (relatedness, belonging, social well-being). This convergence suggests a stable, theoretically grounded core to the flourishing construct despite surface-level definitional variation. The Healthy Minds Framework (Dahl et al., 2020) is broadly convergent with this core: its Purpose dimension corresponds to meaningful engagement, its Connection dimension maps onto the relational well-being element, and its Awareness dimension constitutes a trainable prerequisite for the self-regulatory capacity that underpins autonomous functioning across frameworks. Crucially, however, it adds a dimension, the neurobiological trainability of well-being skills through contemplative practice that the other four frameworks do not address, positioning flourishing as a learnable capacity with measurable neural correlates rather than solely as a psychological outcome of favourable conditions. Divergence was most pronounced in the treatment of virtuous character and moral functioning (emphasized more strongly by VanderWeele than by other frameworks), in the explicit inclusion of physical health (Seligman, 2018; VanderWeele, 2017, versus Keyes, 2002), and in the motivational mechanisms proposed to explain flourishing trajectories (SDT’s basic psychological needs versus PERMA’s structural domain model).

A critical finding from the theoretical synthesis concerns the treatment of flourishing as a state versus a process or way of life. Review of recent theoretical work by Verma et al. (2025), Fowers et al. (2024), and Lomas et al. (2023) revealed growing consensus that flourishing is better conceptualized as an active, ongoing striving for excellence and virtuous engagement consistent with Aristotle’s original eudaimonic vision rather than as a discrete psychological state achieved at a given moment. This distinction has significant implications for measurement (favoring longitudinal and ecological momentary designs over cross-sectional snapshots) and intervention (favoring sustained, habit-forming programs over brief, state-inducing exercises).

A further critical observation concerns the theoretical limitations of each framework when scrutinised in comparative perspective. SDT, which has provided the dominant motivational architecture in this literature, offers a sophisticated account of the conditions under which need satisfaction produces flourishing but remains largely agnostic regarding social-structural determinants: the theory is largely silent on how poverty, discrimination, and structural inequality systematically thwart basic psychological needs, thereby limiting its explanatory power beyond individually calibrated interventions (Vansteenkiste et al., 2020). PERMA, by contrast, offers high accessibility and measurement tractability but has been criticised for treating its five elements as co-equal domains rather than as constructs of differing causal depth, engagement and positive emotion, for instance, plausibly function as proximal markers rather than independent constituents of flourishing (Fowers et al., 2024). Keyes’s two-continua model makes the clinically important distinction between the absence of disorder and the presence of flourishing, yet its operationalisation of social well-being has attracted criticism for conflating individual and collective phenomena within a single measurement scale. VanderWeele’s comprehensive framework uniquely incorporates virtue and moral character as flourishing dimensions, but the normative presuppositions embedded in this claim risk importing culturally specific conceptions of the good life into an ostensibly empirical construct (Rule et al., 2024). Genuine theoretical integration as distinguished from parallel citation of multiple frameworks requires explicit acknowledgment of these incompatibilities and a specification of where different frameworks provide complementary versus competing accounts of the same phenomenon.

The comparison of frameworks across developmental contexts further reveals that no single theory possesses sufficient developmental scope to account for flourishing across the full lifespan. SDT’s basic psychological needs framework has been most extensively validated in educational and workplace contexts (Howard et al., 2024; McAnally and Hagger, 2024), yielding more limited developmental differentiation across childhood, adolescence, and late adulthood. Keyes’s framework was originally developed and validated with adult community samples, and its social well-being subscales have shown lower factorial validity in adolescent populations (Keyes, 2006; Waigel and Lemos, 2023). PERMA has demonstrated the broadest cross-developmental measurement validity, though its sensitivity to the qualitatively distinct developmental challenges of, for example, early childhood or late adulthood remains insufficiently examined. These limitations do not invalidate any framework but collectively argue against the heuristic convenience of selecting a single theoretical lens as the default organizing architecture for lifespan flourishing research, a practice that produces coverage without depth and risks obscuring precisely the cross-framework tensions that are theoretically most informative.

**Figure 2:**
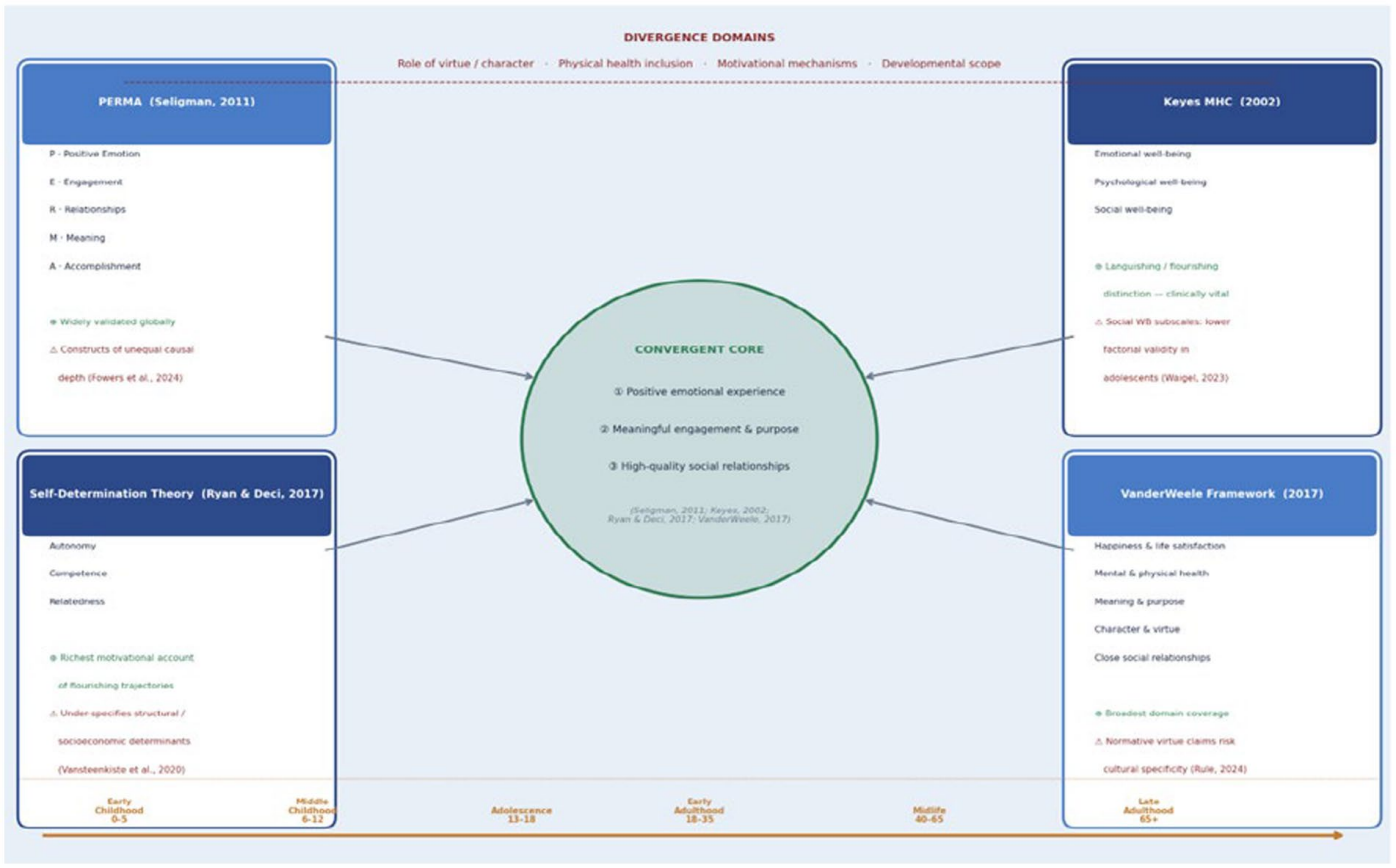
Cross-framework theoretical integration.

Three unresolved conceptual tensions require explicit recognition, as each limits the field’s ability to develop cumulative knowledge regardless of the framework adopted. The first is the construct boundary problem. The four frameworks differ not only in their identification of the components of flourishing, but also in their underlying assumptions about the nature of flourishing itself. PERMA and Keyes conceptualize flourishing primarily as a psychological state, SDT frames it as a condition achieved through motivational processes, whereas VanderWeele’s framework defines it as a normatively grounded, multidimensional achievement spanning several life domains. These are not surface disagreements resolvable by measurement refinement; they reflect genuinely incompatible ontological commitments about what kind of thing flourishing is. A study using the PERMA-Profiler and a study using the VanderWeele Flourishing Index are not measuring the same construct with different instruments; they are measuring different constructs that share a name (Rule et al., 2024). Until this boundary problem is resolved either by theoretical integration or by explicit disciplinary division into sub-fields aggregate claims about flourishing prevalence, predictors, and interventions will remain difficult to interpret. The second tension is the state-versus-process dispute: Fowers et al. (2024) and Lomas et al. (2023) converged on the view that flourishing is better understood as an ongoing orientation toward virtuous engagement than as a state achieved at a point in time, yet all four dominant frameworks operationalise flourishing as a current-state variable susceptible to cross-sectional measurement. This creates a systematic mismatch between theory and method that cannot be addressed without longitudinal and ecological momentary designs as the field’s primary evidentiary standard. The third tension is the universality-versus-cultural-specificity problem: all four frameworks claim cross-cultural applicability, yet each was developed within Western, educated, industrialized contexts, and the cultural variation documented in cross-national validation studies particularly in the relative weighting of individual versus relational flourishing dimensions is treated as measurement error rather than as evidence that the frameworks’ construct definitions may themselves be culturally bounded (Mahamid et al., 2023; Al-Hendawi and Alodat, 2024). These three tensions are not limitations of individual studies; they are structural features of the current theoretical landscape that any integrative synthesis must acknowledge rather than resolve by fiat (Figure 2).

### 4.2 Empirical evidence on developmental variation

Synthesis of empirical evidence across developmental stages revealed systematic variation in the predictors, correlates, and prevalence of flourishing at different life phases. In early childhood and middle childhood, the quality of caregiving relationships emerged as the dominant predictor of flourishing-related outcomes (Mikulincer and Shaver, 2020; Waldinger and Schulz, 2023), with autonomy-supportive parenting and warm teacher-student relationships exerting independent effects. In adolescence, peer relationships, school belonging, and identity formation processes became increasingly salient alongside family relationships, and the role of digital social environments emerged as a significant additional predictor domain (Marciano et al., 2023; Waigel and Lemos, 2023). The PERMA-Profiler demonstrated acceptable psychometric properties in Brazilian (Fernandes et al., 2024), Mexican (Chaves et al., 2023), Qatari (Al-Hendawi and Alodat, 2024), and Arabic-speaking Palestinian (Mahamid et al., 2023) adolescent samples, supporting cross-cultural measurement validity.

In early and middle adulthood, flourishing was most strongly predicted by occupational engagement, romantic partnership quality, purposeful self-investment, and autonomy support in educational and workplace contexts (Verma et al., 2025; Howard et al., 2024). The protective effects of flourishing against subsequent mental disorder documented across three age cohorts in Burns et al. (2022) longitudinal study were consistent across early, middle, and later adulthood, suggesting that the preventive function of flourishing is not confined to any single developmental period. In late adulthood, Fastame et al. (2024) hierarchical regression analysis identified satisfaction with family ties, resilience, and metacognitive efficiency as the strongest predictors of flourishing among adults aged 65–94, with sociodemographic variables (particularly gender) exerting additional significant effects. Carstensen et al. (2020) findings confirmed that older adults maintain positive emotional experience even under conditions of pandemic-related threat, consistent with Socioemotional Selectivity Theory predictions.

Importantly, prevalence estimates varied considerably across developmental stages and cultural contexts. Burns et al. (2022) reported that fewer than 20% of adults in high-income countries met the criteria for flourishing at any given assessment. In contrast, cross-sectional studies involving adolescents and young adults indicated particularly low flourishing rates among post-2012 cohorts exposed to high levels of social media use (Blanchflower and Bryson, 2025). Studies of late adulthood, however, consistently reported higher flourishing rates than those observed in mid-adulthood. This pattern aligns with the well-established U-shaped curve of well-being, while challenging simplistic deficit-oriented accounts of ageing. The cross-framework measurement discordance problem was also empirically visible in the developmental data: studies using the PERMA-Profiler and studies using the VanderWeele Flourishing Index for ostensibly comparable samples produced non-overlapping prevalence estimates, underscoring the construct boundary problem identified. Recent work by Zhang et al. (2026) demonstrate that the successful integration of generative AI tools in educational settings is mediated by motivational dynamics consistent with SDT, the same theoretical architecture that underlies flourishing at the individual level.

What is missing from the developmental evidence base is as informative as what is present. Four gaps are particularly consequential. First, flourishing in early childhood (ages 0–5) is almost entirely unrepresented in the quantitative literature: no included study used a validated flourishing measure with samples below age 6, and the developmental foundations data reviewed here rests on proxies (attachment security, emotion regulation, autonomy-supportive parenting) rather than direct flourishing assessment. Second, the transition periods early-to-middle childhood, adolescence-to-early adulthood, and midlife-to-late adulthood are theoretically critical moments for flourishing trajectories but are underrepresented relative to stable-period assessments; no included study tracked flourishing continuously through a major developmental transition within a single sample. Third, flourishing in marginalized populations including racially and ethnically minoritized individuals, LGBTQ+ people, neurodivergent individuals, and those in low-income or conflict-affected contexts was almost entirely absent from the included sample, limiting the generalizability of all developmental findings. Fourth, the neurobiological substrates of developmental flourishing trajectories how the brain’s reward, social, and self-regulatory systems change across the lifespan in ways that shape flourishing capacity remain unaddressed by the frameworks and measures currently dominant in the field, a gap that the Healthy Minds Framework (Dahl et al., 2020) is positioned to address but has not yet done so within a lifespan developmental design. Where to go next: longitudinal cohort studies spanning at least two major developmental transitions, using multi-instrument flourishing batteries that permit cross-framework comparison within the same sample, and that deliberately oversample the population groups currently absent from the literature, are the highest-priority empirical investments this synthesis identifies for the developmental evidence stream.

### 4.3 Key predictors and moderators across the lifespan

A synthesis of predictors identified across developmental stages revealed several cross-cutting factors that consistently predict flourishing regardless of developmental period. Social relationships and perceived social support emerged as the single most consistent cross-developmental predictor across all included studies, followed by sense of meaning and purpose, trait positive affect and emotional stability, and psychological resilience. At the individual level, resilience, emotional regulation quality, character strengths, and cognitive self-efficacy were identified as robust predictors across studies from adolescence through late adulthood. At the contextual level, autonomy support from caregivers, teachers, supervisors, and digital interfaces emerged as a critical environmental determinant of flourishing across developmental periods, consistent with SDT’s predictions (Ryan and Deci, 2017; McAnally and Hagger, 2024).

Several important moderators of the flourishing–predictor relationship were identified. Cultural context moderated the relative weighting of individual versus relational flourishing dimensions, with collectivist cultural settings showing stronger associations between relational well-being and flourishing outcomes, and individualist settings showing stronger associations with personal autonomy and self-actualization dimensions. Developmental stage moderated the role of digital engagement: positive associations between quality social media use and flourishing were most consistently observed in adolescence (Marciano et al., 2023; Janicke-Bowles et al., 2023), while nuanced patterns emerged in older adulthood where digital literacy and access inequality introduced additional moderating effects. Neurodevelopmental status emerged as a significant moderator of the relationship between educational contexts and flourishing, with neurodivergent learners showing differential responses to standard instructional environments relative to gamified and structured digital alternatives (Jiang et al., 2025). However, the predictor evidence carries important qualifications. Effect sizes for social relationship quality as a flourishing predictor were consistently in the moderate range (r = 0.30–0.45) across included observational studies, but causal inference is constrained by the cross-sectional design of 61% of the included observational sample; reverse causation that flourishing promotes relationship quality rather than the reverse cannot be ruled out from existing data. The association between autonomy support and flourishing, while consistent across educational and workplace contexts (Howard et al., 2024; McAnally and Hagger, 2024), has been tested almost exclusively in Western samples, and the extent to which autonomy operationally defined as self-endorsement and volitional engagement carries the same meaning and the same relationship to flourishing in high-power-distance cultural contexts remains an empirical question rather than an established finding.

What is missing from the predictor and moderator evidence is a systematic test of predictor specificity: the field has documented that social relationships, meaning, autonomy support, and resilience all predict flourishing, but it has not established the relative magnitude of these predictors in competition with one another within multi-predictor designs, nor whether their relative importance shifts across developmental stages in ways predicted by any of the theoretical frameworks reviewed. The moderator evidence for cultural context is suggestive but methodologically thin: most cultural moderation findings come from between-study comparisons rather than within-study cross-cultural designs, making observed cultural differences vulnerable to confounding by study design, measurement instrument, and sample characteristics. Contemplative skills attentional regulation, compassion, insight, and purpose as operationalised in the Healthy Minds Framework (Dahl et al., 2020) were not tested as predictors in any included study, representing a gap that limits the field’s ability to distinguish environmentally mediated from individually cultivated routes to flourishing. Where to go next: pre-registered within-person longitudinal studies using ecological momentary assessment of flourishing alongside competing predictors relationship quality, meaning, autonomy, contemplative practice engagement would permit the causal and relative-magnitude questions to be addressed in a single design, while deliberate cross-national replication with locally adapted instruments is required before cross-cultural moderator claims can be treated as established.

### 4.4 Evidence on flourishing interventions

The synthesis of intervention evidence yielded several robust findings. Positive psychology interventions (PPIs) demonstrated moderate to large effect sizes for improving subjective well-being, positive affect, and several PERMA dimensions across meta-analyses of randomized controlled trials, with sustained effects at 3- to 6-month follow-up assessments (Hendriks et al., 2020). Developmental tailoring emerged as a critical moderator of intervention efficacy: personalized, pre-assessed PPIs that targeted specific PERMA dimensions showing the greatest developmental salience at a given life stage consistently outperformed generic positive education curricula. PERMA-based school interventions in China demonstrated significant improvements in psychological resilience, positive coping tendencies, and multidimensional well-being in junior high school students (Zhang et al., 2025), while teacher flourishing programs yielded multiplicative benefits through improved implementation of well-being-promoting pedagogies. Mindfulness-based and contemplative interventions, operationalised within the Healthy Minds Framework (Dahl et al., 2020), constitute a parallel and rapidly expanding evidence stream not fully captured by the PPI literature reviewed above. Goldberg et al. (2021) meta-analysis of smartphone-delivered mindfulness programmes found significant effects on well-being, stress, and depression relative to active controls, while Goldberg et al. (2022) systematic review of 44 mindfulness meta-analyses confirmed robust, replicable effects across delivery formats, populations, and outcome domains. These findings are particularly relevant for digital learning contexts: unlike most PPIs, which are designed for synchronous group or individual delivery, mindfulness-based interventions have been validated extensively in asynchronous digital formats, with effect sizes comparable to in-person delivery, making them a viable and under-utilized resource for flourishing promotion in technology-mediated educational environments.

What is missing from the intervention evidence is considerable. The active ingredient problem remains unresolved: neither PPI meta-analyses nor mindfulness reviews have established which specific components drive effect gratitude practice, meaning-making, attentional training, compassion cultivation, or social connection exercises for which populations and at which developmental stages. This is not a trivial methodological gap; without active-ingredient data, intervention developers cannot make principled decisions about which elements to include or scale, and clinicians cannot select components matched to individual client profiles (Shoshani and Slone, 2013). Intervention evidence for the developmental bookends early childhood and late adulthood is particularly thin: of the seven intervention studies included in the synthesis, nontargeted children under age 8 or adults over 75 as primary samples, leaving the developmental stages with the least existing flourishing infrastructure also the least supported by intervention evidence. Most included trials were also conducted in high-income, WEIRD educational or clinical settings, and only one included trial the China-based PERMA school intervention (Zhang et al., 2025) represented a non-Western population, precluding conclusions about cross-cultural intervention transportability. Where to go next: dismantling trials that isolate active components within multi-element PPIs; replication studies in non-WEIRD contexts with locally adapted protocols; developmental extension of both PPI and mindfulness-based paradigms into early childhood and late adulthood samples; and systematic evaluation of digital delivery formats building on the Goldberg et al. (2021, 2022) evidence base as a scalable vehicle for reaching populations that in-person interventions fail to reach.

## 5 Discussion and conclusion

### 5.1 Integrative discussion

The present integrative review synthesized theoretical and empirical evidence pertaining to psychological flourishing across the human lifespan, drawing on six major theoretical frameworks, 62 empirical and theoretical sources, and evidence spanning early childhood through late adulthood. The findings cohere around several major themes that merit detailed discussion.

First, psychological flourishing is genuinely multidimensional, and no single theoretical framework fully captures its complexity. The convergence of PERMA, Keyes’s two-continua model, SDT, and VanderWeele’s comprehensive framework on three core components—positive emotional experience, meaningful engagement, and high-quality social relationships suggests that these dimensions constitute a robust empirical and theoretical core. However, the divergence between frameworks on the role of virtue, physical health, and moral functioning indicates that current dominant models remain incomplete. Fowers et al. (2024) and VanderWeele (2017) made a compelling case that character and virtue deserve reinstatement as integral flourishing dimensions, not merely as correlates or antecedents—a position supported by longitudinal evidence linking virtuous functioning to sustained life satisfaction and health outcomes in adulthood (McAdams and Logan, 2023).

Second, the developmental perspective adopted in this review revealed that flourishing is not a static trait but a dynamic developmental accomplishment whose conditions, challenges, and manifestations shift meaningfully across the lifespan. Theoretical frameworks developed predominantly with adult samples require systematic developmental extension, and intervention research must be calibrated to the motivational, relational, and environmental realities of specific developmental stages. The evidence reviewed supports a lifespan developmental model of flourishing in which early relational experiences establish fundamental psychobiological capacities for need satisfaction, middle developmental periods provide critical opportunities for identity-based flourishing through education and occupation, and late adulthood draws on accumulated relational resources and adaptive meaning-making to sustain flourishing in the face of biological decline.

The findings of this review extend far beyond the academic research community. The importance of psychological flourishing is not merely a theoretical concern, as fewer than one in five adults in high-income countries are currently considered to be flourishing (Keyes, 2002; WHO, 2022). The consequences of this deficit including higher rates of mental disorders, lower productivity, poorer physical health, and reduced civic engagement are experienced by healthcare systems, educational institutions, workplaces, families, and individuals across the lifespan. The science reviewed here is not merely descriptive of this reality; it identifies modifiable determinants at the individual, relational, institutional, and structural levels that make flourishing a tractable public goal. The question of what can be done has concrete, evidence-based answers for clinicians, educators, policymakers, and individuals alike, answers developed in Section 5.4. The question of who should care extends to all those whose professional or personal remit touches human development, mental health, or social welfare which is to say, to an audience far broader than the academic psychology community to which this literature has historically been addressed.

### 5.2 Theoretical implications

The integrative synthesis suggests several theoretical refinements and extensions. First, existing lifespan flourishing models would benefit from formal specification of the mechanisms through which early relational experiences translate into adult flourishing dispositions a translation that likely involves both psychological (emotional regulation, meaning making) and neurobiological (stress regulation, allostatic load) pathways not yet fully articulated in dominant frameworks. Second, the growing evidence base on digital and AI-mediated flourishing calls for the explicit incorporation of digital context into comprehensive flourishing frameworks, as VanderWeele (2017) pathway-based model developed before the maturation of digital ecology research does not yet systematically address the digital domain. Third, the cultural variation documented in flourishing predictors and moderators calls for more explicit theoretical specification of culturally universal versus culturally contingent dimensions of flourishing, a distinction that has both theoretical and practical implications for the global dissemination of flourishing science.

### 5.3 Practical implications

The evidence synthesized in this review carries concrete implications for four distinct stakeholder communities, each with a different answer to the questions of who should care, why it matters, and what can be done. For clinicians and mental health practitioners, the central implication is that clinical assessment and intervention goals need to extend beyond symptom reduction. Flourishing is a clinically measurable and modifiable target distinct from symptom absence. Burns et al. (2022) longitudinal evidence that flourishing at baseline predicts significantly lower rates of depression and anxiety across subsequent decades establishes flourishing as a clinically actionable target not merely a philosophical aspiration. A patient who no longer meets diagnostic criteria for depression but remains in Keyes (2002) languishing category faces substantially elevated risk of relapse and impaired functioning. Practitioners should therefore routinely screen for the presence of flourishing positive relationships, meaning, engagement, and purpose alongside standard psychopathology assessment, and should be equipped with evidence-based positive psychology interventions (PPIs) that move clients toward complete mental health rather than simply away from disorder. Hendriks et al. (2020) meta-analytic findings that multi-component PPIs achieve moderate-to-large effect sizes at 3–6-month follow-up provide a credible evidence base for this clinical shift. What remains unknown or debated: which specific PPI components drive effects, for whom, and at which developmental stage remains poorly understood; most intervention trials were conducted with non-clinical adult samples, leaving the evidence base for clinical populations, children, and older adults underdeveloped. The optimal measurement instrument for clinical screening is unresolved (Diener et al., 2010): the Keyes MHC-SF, PERMA-Profiler, and Diener Flourishing Scale yield partially divergent profiles for the same individual, and no instrument has been validated for routine clinical use across the lifespan. Whether flourishing-promoting interventions reduce long-term relapse rates, the most clinically consequential question has not been tested in randomised designs with adequate follow-up. What to prioritize: screen for languishing as a routine component of clinical assessment alongside disorder criteria; integrate evidence-based PPIs into stepped-care models proportionate to need; advocate within professional bodies for the inclusion of flourishing outcomes in treatment guidelines; and support trials that extend PPI evidence into clinical, paediatric, and geriatric populations with long-term follow-up.

For educators and school leaders, the evidence is both compelling and immediately actionable. Schools are among the most consequential flourishing environments across the lifespan: the quality of teacher–student relationships, the extent to which curricula support autonomy and competence, and the degree to which students experience belonging and meaning are all robust predictors of flourishing at school age and beyond (Howard et al., 2024; Seligman et al., 2009). The practical implication is not to add a well-being “subject” to an already full curriculum but to embed flourishing-oriented pedagogical principles choice, rationale, acknowledgment of student perspectives, and genuine relatedness into routine instructional practice across all subjects and stages. Longitudinal evidence from the Harvard Study of Adult Development (Waldinger and Schulz, 2023) demonstrates that the relational foundations laid in school-age environments have measurable consequences for adult health and life satisfaction decades later, making investment in relational school cultures a matter of long-term public health, not merely educational aspiration. Whether school-based flourishing gains transfer into sustained adult flourishing, or whether they reflect short-term state improvements that fade without continued support, is largely unresolved due to the absence of long-term follow-up in school intervention research. The mechanisms through which teacher flourishing influences student flourishing have been theorized but not experimentally isolated. How to adapt flourishing-promoting pedagogies for neurodivergent learners, students from low-income backgrounds, and culturally diverse classrooms without imposing WEIRD-derived norms remains an open and pressing question. What to prioritize: embed autonomy-supportive instructional practices across subjects rather than adding discrete well-being programmes; invest in teacher well-being as a lever for student flourishing; track student flourishing outcomes longitudinally alongside academic attainment; and commission school-based research that follows students into adulthood to test whether school-age gains persist.

For policymakers and public health agencies, the most pressing implication concerns the distribution of flourishing. The WHO (2022) finding that fewer than 20% of adults in high-income countries meet criteria for flourishing—with rates as low as 5–10% in low-income contexts constitute a population-level deficit with measurable consequences for economic productivity, healthcare utilization, and social cohesion. Because structural determinants socioeconomic position, housing stability, access to education, community safety, and equitable employment systematically shape the conditions under which basic psychological needs can be met, flourishing cannot be meaningfully promoted through individually targeted programmes alone. Policy investment in the environmental preconditions of flourishing safe and relationally rich early childhood environments, accessible higher education, meaningful work, age-friendly community infrastructure, and reduction of structural poverty and discrimination represents the highest-leverage intervention available at population scale. Flourishing-informed policy does not require a new budget line; it requires reframing existing investments in health, education, housing, and labor as flourishing infrastructure.

For individuals and families, the review translates into a set of empirically grounded orientations rather than prescriptions. Across developmental stages, the evidence consistently points to three modifiable levers of personal flourishing: the investment in and cultivation of close, reciprocal relationships (Waldinger and Schulz, 2023; Fastame et al., 2024); active engagement with meaningful goals and activities that draw on one’s strengths (Seligman, 2011; VanderWeele, 2017); and the pursuit of environments whether educational, occupational, or community-based that support autonomy and a sense of competence (Ryan and Deci, 2017). Importantly, flourishing is not a fixed trait possessed by some and absent in others; the developmental evidence reviewed here demonstrates that it is cultivable across the entire lifespan, including in late adulthood where the “well-being paradox” (Carstensen et al., 2020) reveals that adaptive meaning-making and selective relational investment can sustain flourishing even in the presence of physical decline.

This study provides findings for Researchers. Three irresolvable conceptual tensions currently constrain cumulative knowledge—the construct boundary problem, the state-versus-process dispute, and the universality-versus-cultural-specificity problem and no single theoretical framework has adequate developmental scope to account for flourishing across the full lifespan. Quality appraisal found that 32% of observational studies received partial adequacy ratings predominantly due to inadequate confounding control and attrition reporting, and 43% of intervention studies were rated partial due to absent allocation concealment and limited fidelity reporting, establishing a systematic evidence quality deficit that narrows the conclusions researchers can defensibly draw. What remains unknown or debated: the causal mechanisms linking developmental experience to adult flourishing dispositions are theorized but not empirically isolated; sensitive periods for flourishing intervention across the lifespan have not been identified through longitudinal designs; the relative explanatory contributions of hedonic versus eudaimonic dimensions at different life stages remain contested; and the cross-cultural generalizability of all four major frameworks is contested but under-tested in non-WEIRD populations. What to prioritize: pursue pre-registered longitudinal cohort studies that track flourishing from childhood through late adulthood using invariant measures; develop and validate a single, developmentally sensitive flourishing instrument capable of detecting stage-appropriate variation; explicitly design studies to test cross-framework predictions against the same dataset, enabling competitive rather than parallel framework evaluation; invest in participatory and community-based research with structurally disadvantaged populations to generate evidence that does not simply replicate WEIRD findings in new settings; and treat the three conceptual tensions identified in this review as testable empirical hypotheses rather than background assumptions.

### 5.4 Limitations of the review

Several limitations of the present review warrant acknowledgment. The integrative review design, while enabling broad synthesis, precludes the quantitative precision of meta-analytic effect size estimation. Most included studies were conducted in high-income, WEIRD (Western, Educated, Industrialized, Rich, Democratic) national contexts, limiting the global generalizability of conclusions. The evidence base on digital and AI-mediated flourishing while expanding rapidly remains relatively young and is dominated by cross-sectional designs that preclude strong causal inference. Measurement heterogeneity across included studies complicated direct comparison of findings, and the absence of a universally adopted, developmentally validated flourishing measure remains a significant methodological constraint. Future syntheses would benefit from a pre-registered systematic review protocol, formal inter-rater agreement procedures for study selection, and quantitative meta-analytic methods applied to specific, well-operationalized flourishing subdomains.

### 5.5 Directions for future research

The following priorities are organized by stakeholder group, reflecting the argument developed that the questions most urgently needing resolution differ across the communities that flourishing science is now reaching. For researchers, the highest-priority investments are: (a) pre-registered longitudinal cohort designs that track the same individuals from childhood through late adulthood using a developmentally invariant or developmentally calibrated flourishing measure, specifically designed to test the competing developmental predictions of PERMA, SDT, and VanderWeele’s framework against a single dataset; (b) competitive rather than parallel framework evaluation studies that pit the specific predictions of two or more frameworks against each other within a single design rather than citing multiple frameworks as complementary without specifying their differential predictions; (c) theory-driven cross-cultural replication studies in non-WEIRD contexts, explicitly designed to test whether construct definitions hold or require revision when cultural presuppositions about the primacy of individual versus relational flourishing differ; and (d) participatory research designs that position structurally disadvantaged communities, low-income, racially marginalised, LGBTQ+, neurodivergent, and refugee populations as co-investigators rather than study subjects, enabling flourishing constructs to emerge from rather than be imposed upon their experience. For clinicians and healthcare systems, the priority is evidence on which PPI components are active ingredients versus inert for which client populations, examined in trials that include clinical samples with adequate long-term follow-up and that use flourishing not just symptom reduction as the primary outcome. For educators and school systems, the priority is longitudinal evaluation of school-based flourishing programmes that tracks students into adulthood, enabling the field to determine whether school-age gains are durable or require environmental scaffolding to persist. For policymakers, the priority is natural experiments and quasi-experimental evaluations of structural interventions housing, employment, early childhood investment that measure flourishing outcomes alongside traditional economic and health metrics, generating the cost-effectiveness evidence base that population-level flourishing policy currently lacks. Across all groups, the single unresolved question with the highest stakes is the state-versus-process dispute: if flourishing is an orientation or way of life rather than a quantifiable state, the field’s entire measurement infrastructure requires fundamental reconsideration, and this question should be treated as an empirical priority rather than a philosophical aside.

### 5.6 Conclusion

Psychological flourishing is one of the defining aspirations of the human condition and one of the most consequential targets of contemporary psychological science. The present integrative review has synthesized evidence from positive psychology, developmental psychology, educational psychology, and related disciplines to construct a comprehensive account of how flourishing unfolds, varies, and can be cultivated across the human lifespan. Several overarching conclusions merit emphasis.

Flourishing is genuinely multidimensional: its core involves positive emotion, meaningful engagement, and social relatedness, with additional dimensions of virtue, health, and autonomy recognized across frameworks. Flourishing is fundamentally developmental: its determinants, challenges, and manifestations shift systematically across the lifespan, necessitating stage-sensitive theory, measurement, and intervention. Flourishing is inherently relational and contextual: it is constituted in and sustained by relationships, communities, institutions, and increasingly digital environments, not merely within individual minds. These conclusions carry implications that extend well beyond the boundaries of psychological science. For the clinician, they reframe the goal of treatment from illness to health. For the teacher, they reframe the purpose of education from attainment to flourishing. For the policymaker, they reframe social investment from cost management to the cultivation of human potential. For the individual navigating midlife difficulty, raising a child, caring for an ageing parent, or facing retirement, they provide empirically grounded reassurance that flourishing is not the province of the fortunate few but a developmental possibility across the full arc of human life and a legitimate, evidence-supported goal for science, practice, and society alike.

The studies by Jiang et al. (2025) and Zhang et al. (2026) collectively illustrate that digital and AI-mediated educational environments represent a new frontier of flourishing science, with the design principles of gamification and autonomy-supportive AI integration emerging as promising levers for cultivating the engagement, emotional regulation, and autonomous motivation that are the proximal psychological preconditions for flourishing. As artificial intelligence, digital learning, and immersive technology become ever more deeply woven into the fabric of human development across the lifespan, ensuring that these technologies are designed in accordance with flourishing science and that their benefits are equitably distributed becomes not merely a research priority but a moral imperative. The science of flourishing must rise to meet this challenge.
